# Gossypiboma Causing Mechanical Intestinal Obstruction: A Case Report

**DOI:** 10.1155/2012/543203

**Published:** 2012-10-24

**Authors:** Akin Aydogan, Seckin Akkucuk, Ibrahim Yetim, Orhan Veli Ozkan, Murat Karcioglu

**Affiliations:** ^1^Department of General Surgery, Faculty of Medicine, Mustafa Kemal University, Serinyol, 31100 Hatay, Turkey; ^2^Department of General Surgery, Faculty of Medicine, Sakarya University, Adapazari, 54100 Sakarya, Turkey; ^3^Department of Anesthesiology, Faculty of Medicine, Mustafa Kemal University, Serinyol, 31100 Hatay, Turkey

## Abstract

*Introduction*. Gossypiboma (GP) is a term used to express the mass resulting from forgotten cotton sponge in operations. Rarely, a transmural migration may occur into the gastrointestinal lumen without creating any defect by GP. Laparotomy or endoscopic removal may be required, by the way it can be taken out of the body itself by intestinal ways. In this study, we reported a case of mechanical intestinal obstruction causing GP. *Case*. The fifty-one-year-old female patient admitted to the emergency department with the complaints of mechanical intestinal obstruction and had a history of open cholecystectomy 20 years ago. There were the findings of intestinal obstruction in abdominal plain radiography and computerized tomography. The sponge that obstructed the lumen completely 40 cm proximal to the ileocecal valve was identified in the laparotomy with the diagnosis of brid ileus. The small intestine was closed over double-fold after removal of sponge. Transmural migration of abdominal-remained sponge was thought to be occurred without creating a defect after cholecystectomy. Postoperatively, the patient was discharged without having any problems at 4th day of hospitalization. *Conclusion*. Although it is a rare situation in routine clinical practice, GP should be considered as a differential diagnosis in the patients who had a diagnosis of mechanical intestinal obstruction, and laparotomy was applied before. As GP may lead to situations which cause mortality, all precautions should be taken to prevent it.

## 1. Introduction

Gossypiboma  (GP) is a term used to express the resulting in mass by the remained nonradiopaque cotton sponge or forgotten abdominal compress device in operations. “Textiloma” or “cottonoid”  is also used for expression  [1]. Declaration is not able to be done exactly due to having a medicolegal  problem for hospitals  and  surgeons. GP  may lead to clinical situations causing mortality, such as ileus, abscess, fistula, and bowel necrosis. Rarely, GP is able to do transmural migration without creating any defect into the gastrointestinal lumen [2, 3]. Laparotomy or endoscopic removal may be required, by the way it can be taken out of the body itself by intestinal ways. The diagnosis may be difficult because of its nonspecific imaging features and various clinical appearances such as ileus and abscess formation. In this study, we reported a case of mechanical intestinal obstruction causing GP.

## 2. Case

**Figure 1:**
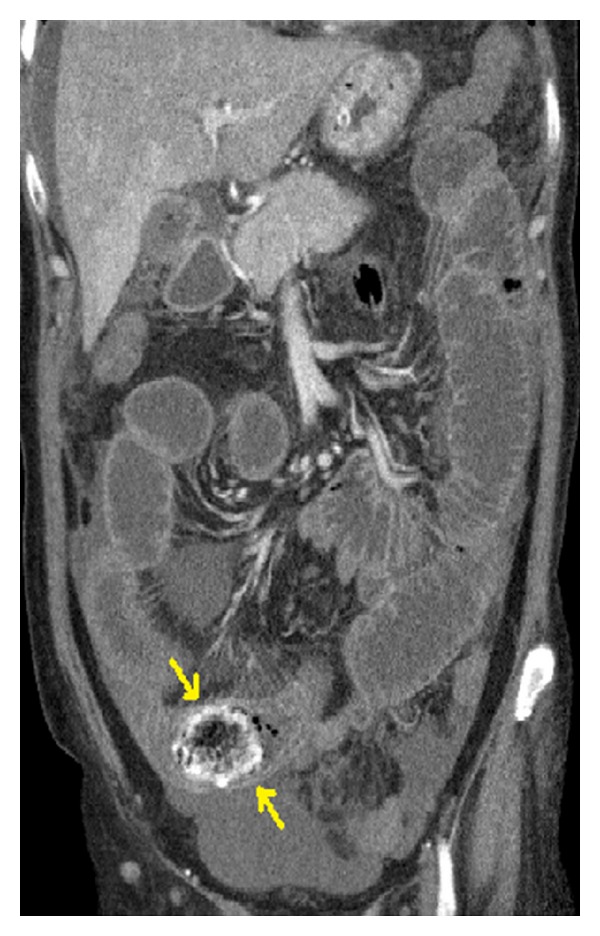
Obstructive lesion in terminal ileum and proximal dilatation on abdominal CT.

**Figure 2:**
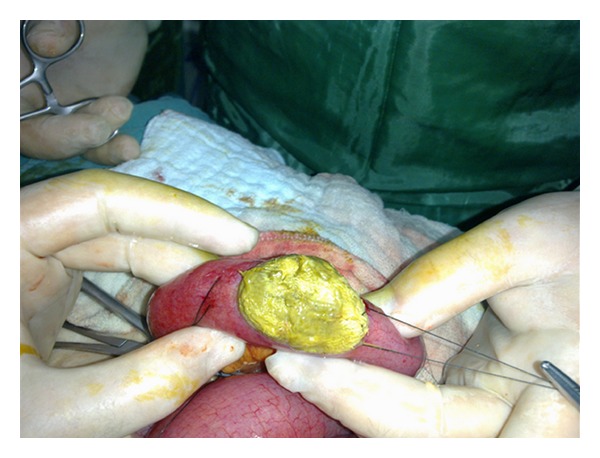
GP's appearance in an enterotomy-applied ileum during operation.

**Figure 3:**
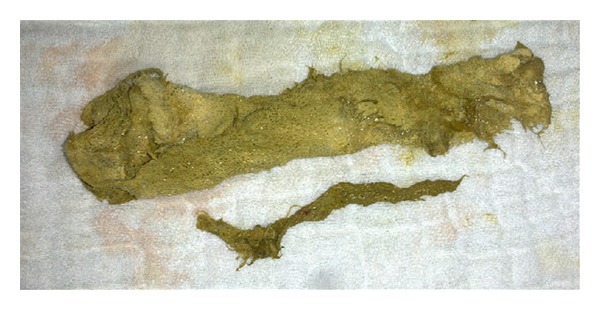
An opened form of sponge extracted from ileum.

The female patient, fifty-one-year-old, admitted to the emergency department with the complaints of mechanical intestinal obstruction, such as nausea, vomiting, abdominal pain, abdominal swelling, and obstipation. The patient also had a history of open cholecystectomy 20 years ago. On physical examination, common abdominal distention and right subcostal incision were seen together. Hyperreactivity of intestinal sounds was heard in auscultation. There was no defense and rebound on palpation. And also, there was no abnormality in laboratory investigations. Air-liquid levels were seen on abdominal plain radiography. On abdomen computed tomography (CT), there was a dilatation in the small intestine at the level of terminal ileum, and there was an obliterating lesion at terminal ileum (Figure 1). The patient was followed-up with the diagnosis of brid ileus. The patient, who did not exhibit any improvement on clinical symptoms and mechanical intestinal obstruction in radiological investigations, was operated at the 2nd day of hospitalization. A mass that obstructed the lumen completely 40 cm proximal to the ileocecal valve was identified as 5 cm in the laparotomy. The removed mass was detected as a sponge by enterotomy (Figures 2 and 3). The small intestine was closed over double-fold after removal of sponge. In following exploration, the liver was found to be highly adherent to duodenum at the region of gallbladder. These findings demonstrated that abdominal-remained-sponge after cholecystectomy migrated with transmural course without creating any defect to duodenum and caused mechanical intestinal obstruction in ileum because of its diameter. Postoperatively, the patient was discharged at 4th day without having any problems.

## 3. Discussion

The risk factors such as emergency operations, exigency of applying unexpected surgical procedure, obese patient, poor organization, quick sponge count, failure in sponge counting, long operations, unstable patients and operations of the assistants were identified for abdominal-forgotten-sponge [4, 5]. GP may cause abdominal distention, ileus, pain, tenesmus, palpable mass, vomiting, weight loss, diarrhea, abscess formation, and fistula. It led to ileus in this patient. 

Radiologic diagnosis of sponge is difficult without a radiopaque marker. It can be detected directly in X-rays when radiopaque marker is available. Especially in chronic cases, because of the probability of imitating the malignant mass, ultrasonography (USG), CT, or magnetic resonance imaging may be necessary. It is able to give us confirmatory evidence, in case we suspected a sponge remained in abdomen. In this patient, ileus was determined by taking abdominal X-ray and CT. In CT, spongiform structure containing the cystic lesions, air bubbles, hyperdense capsule, and spotted calcifications can be seen characteristically inside GP. The mass has got the features in the form of demarcated, hypoechoic sided, strong acoustically shaded, undulated internal echogenic focus in USG [6, 7]. In this patient, there was no typical image of the GP. This situation was suggested to be due to the absence of radiopaque substance in sponge, and a long time over 20 years had passed from the first operation. 

A sponge that is remained in abdomen may result in perforation of intestine or other organs, obstruction, sepsis, and even death. Additionally, it may migrate to ileum, stomach, colon, and bladder without creating any defect on surface of those. Migration to intestine may cause partial or complete obstruction and bleeding due to erosions of the mucosa. Besides, migration to bladder may result in urinary problems. Some patients can be detected incidentally after staying asymptomatic for many years. Another possibility is excretion of the sponge with peristaltic movements via rectum spontaneously, after its migration to any part of intestine [8]. Defecation of two sponges rectally has even been reported [3]. Foreign body may be removed by endoscopic and laparoscopic ways or with laparotomy, when GP was diagnosed. In this patient, GP was removed by laparotomy because of signs of ileus.

Although GP is a rare situation in routine clinical practice, it should be considered as a differential diagnosis in the patients who had a diagnosis of mechanical intestinal obstruction and laparotomy was applied before. As GP may lead to situations which cause mortality, all precautions should be taken to prevent it. This situation, depending on human fault, can be minimized by staff training, even though it is not prevented entirely.
